# The effect of gender on end‐user preferences for yam quality descriptors

**DOI:** 10.1002/fsn3.2986

**Published:** 2022-08-09

**Authors:** Liticia Effah‐Manu, Faustina D. Wireko‐Manu, Jacob K. Agbenorhevi, Bussie Maziya‐Dixon, Ibok N. Oduro, Theresa Y. Baah‐Ennum

**Affiliations:** ^1^ Department of Food Science and Technology Kwame Nkrumah University of Science and Technology Kumasi Ghana; ^2^ Department of Food Science and Technology Ho Technical University Ho Ghana; ^3^ Postharvest and Nutrition Laboratory International Institute of Tropical Agriculture (IITA) Ibadan Nigeria; ^4^ Department of Planning Kwame Nkrumah University of Science and Technology Kumasi Ghana

**Keywords:** end‐user, ethnicity, gender, preference, yam

## Abstract

Improving the effectiveness of breeding programs could be achieved by breeding for consumers' preferences. The relationship between gender and consumer preferences for yam was studied using purposeful sampling for key informants, focus group discussions (FGDs), and individual interviews. Data from qualitative interviews were transcribed and quantitative data analyzed using SPSS (version 20). Cramer's *V* (Phi) and logistic regressions were used to explain the observations. Males prioritized income‐generating varieties over females, while females ranked tuber morphological characteristics high. Sex‐based preference for yams showed that moldability and sweet taste are preferred by both females and males. Moderately hard boiled yams and shelf stable food products are liked by males, whereas aroma is rated highly by females. Males described yams that have a longer digestion time as “heavy” and preferred such varieties. Watery tubers and tubers not turning brown are important characteristics during processing. The Phi of 0.157 and 0.163 for sex and ethnic groups show moderate to strong associations between choices for descriptors by females, males, and various ethnic groups. These findings imply that gender disaggregation of information on preference for yam quality descriptors should be considered in future yam breeding programs as it may improve adoption rate and enhance food security.

## Graphical abstract

Sweet taste and mouldability are key quality indicators liked by both females and males. Males prefer yams with longer digestion time while females like aroma of yam. Varieties that are not watery and do not brown are important characteristics during processing.

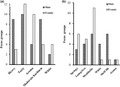

## 1 INTRODUCTION

Yam is a major economic crop in Ghana and has contributed significantly to the economy by meeting household food needs and foreign exchange earnings. In West and Central Africa, yams contribute to the income and food of more than 60 million people (Asiedu & Sartie, 2010). The most important yam species cultivated in Ghana are *D. rotundata (poir)*, *D. alata* (water yam), *D. cayenensis* (yellow yam), *D. trifida* (cushcush yam), and *D. dumetorum* (bitter yam). Ghanaian consumers are, however, very conversant with two varieties, *D. rotundata* and *D. alata,* which are mainly found in the markets (Demuyakor et al., 2013). Generally, in the subregion, yam gives people various opportunities to reduce poverty levels, improve nutrition, and enhance food security (FAO, 2019). In Ghana, yams are eaten as *ampesi* (boiled), *fufu* (pounded), *eto* (mashed), roasted, fried, and *wasawasa* (steamed). Due to the importance of yam to the economic and social lives of Ghanaians, research is ongoing on the accessibility of planting materials, food demand patterns, the socio‐economic contribution of yam, and gendered access to productive resources (Ankrah et al., 2020).

The role of the consumer is important and needs consideration in breeding programs. This is because breeding for consumer preferences (Sanginga & Mbabu, 2015) helps in improving yam production and usage. Studies have, therefore, been conducted into preferences of yams by Aboagye et al. (2015), which were largely dependent on maturity period and ability to withstand biotic and abiotic stresses. In terms of yam research investment, Mignouna et al. (2020) show that the adoption of yam minisett technique (AYMT), nematode‐resistant cultivars (NRC), and crop management postharvest practices (CMPP) would be beneficial as they are less responsive to changes in cost than to adoption rate. Hence, efforts to involve all stakeholders (producers, processors, and consumers) in the value chain, as well as research into gender dimensions, are critical for the production and use of new yams. Bloodhart and Swim (2020) show that the concept of gender is an important role in sustainable consumption and gives a better understanding of consumption patterns. In the study of preferences for food trees in Kenya, it was realized that constraints to the utilization of food trees were gendered (Gachuiri et al., 2022).

According to Löckenhoff et al. (2014), gender refers to the roles and responsibilities of men and women that are created in our societies, cultures, and families. Gender forms part of the broad socio‐cultural context, which includes other important factors such as age, ethnic group, and poverty level (UNFPA, 2011). Furthermore, the preferences of yam consumers are directly linked to the quality expectations that comprise their physical, chemical, and, to a large extent, gender characteristics (Ankrah et al., 2020), which evolve from some socio‐cultural or socio‐economic attributes (Arsel et al., 2015). In this work, “gender parameters” refer to important factors that include sex at birth, ethnicity, age, and occupation that may affect preference for yams by consumers. It is believed that understanding the dynamics of these parameters on the selection of quality descriptors will enhance yam consumption and the adoption of new yam varieties. Disaggregating data based on gender parameters may have a direct effect on food preferences and consumption. It will also help to shape decisions on yam production and processing Doss, (2015), and thus improve food security in Ghana, the subregion, and the world at large. Yam producers will have adequate information to boost their production, while breeders can breed new varieties that will have a high adoption rate.

Food quality is a key determining factor in consumer acceptance and purchase. In recent years, development practitioners have increasingly become concerned about the role of gender in many aspects of agricultural growth. However, consumer behavior is linked to gender schemas. According to Mathes (2019), differences in consumer behavior result from the psychological well‐being of the individual. Similarly, gender could directly affect consumer behavior toward the selection of yams for consumption. The adoption of modern maize varieties has been attributed to gender‐linked differences (Doss et al., 2015). According to Aboagye et al. (2015), the sex of farmers has a great influence on yam production in Ghana. Subsequently, ethnic differences in dietary patterns have been discovered in adolescents in two ethnic groups in Malaysia and China (Abdullah et al., 2016). McNamara and Wood (2019) also found that age and gender‐differentiated taboos affected the intake of certain foods which are central to the Tajik diet. In terms of decision making, Galiè et al. (2020) also concluded that the woman's limited decision‐making power had significant effect on nutritious diet (milk intake) of the entire family. These findings point to the fact that gender could be an important factor in yam preference and consumption.

Factors, such as access to, control over, and utilization of resources, have been major indicators of gender differences (Kassie et al., 2015; Wekwete, 2013). As realized in the work by Tanellari et al. (2014), household heads also have a significant influence on adoption of varieties. Mugonolaa et al. (2013) noted that in fertilizer applications for improved yield, male‐headed households have a higher probability of participation in fertilizer applications than female‐headed households. In the adoption of hybrid seeds, Namonje‐Kapembwa and Chapoto (2016) found that female farmers are less likely to adopt or use fertilizer on their farms compared to their male counterparts. In terms of quality preferences, breeding for new cassava varieties, for example, has been modified with input from female processors who want “easy to peel” varieties (Wossen et al., 2017). Additionally, in linking cassava traits to end‐user preferences by Bechoff et al. (2017), gender was found to be a critical socio‐economic factor that breeders could consider and adapt to maximize income, health, and food security. These findings suggest the importance of gender in the study of yam adoption and end‐user quality preferences. However, there is a paucity of information on gender‐based quality descriptors to inform the 21st century breeder. Information provided in the work could form the basis for a change in breeding priorities to assess the feasibility of selecting such quality traits. The study, therefore, identifies the descriptors of the quality of yam for cultivation and processing of two main food products, *fufu* (pounded) and *ampesi* (boiled) by gender disaggregation of data.

## 2 METHODS

### 2.1 Study location and sample characteristics

The descriptors of the quality of yam‐based foods could be obtained in areas where yam is grown and consumed in significant quantities (Brong Ahafo and Northern regions). The Brong Ahafo region was chosen for the study due to its high production (39% vs. 25% in the Northern region) (SRID, 2011). The region lies between longitude 00 15′ E‐30 W and latitude 80 45′ N‐70 30′ with an estimated population of 2,282,128. The populations of Sene West District, Kintampo municipality, and Techiman East District are, respectively, 57,734, 95,480, and 59,068 (Ghana Statistical Service, 2012). Currently, the creation of new regions has put all these districts under the Bono East Region. According to the census, many households in these districts are involved in agriculture, especially crop farming.

### 2.2 Sampling and samples

**FIGURE 1:**
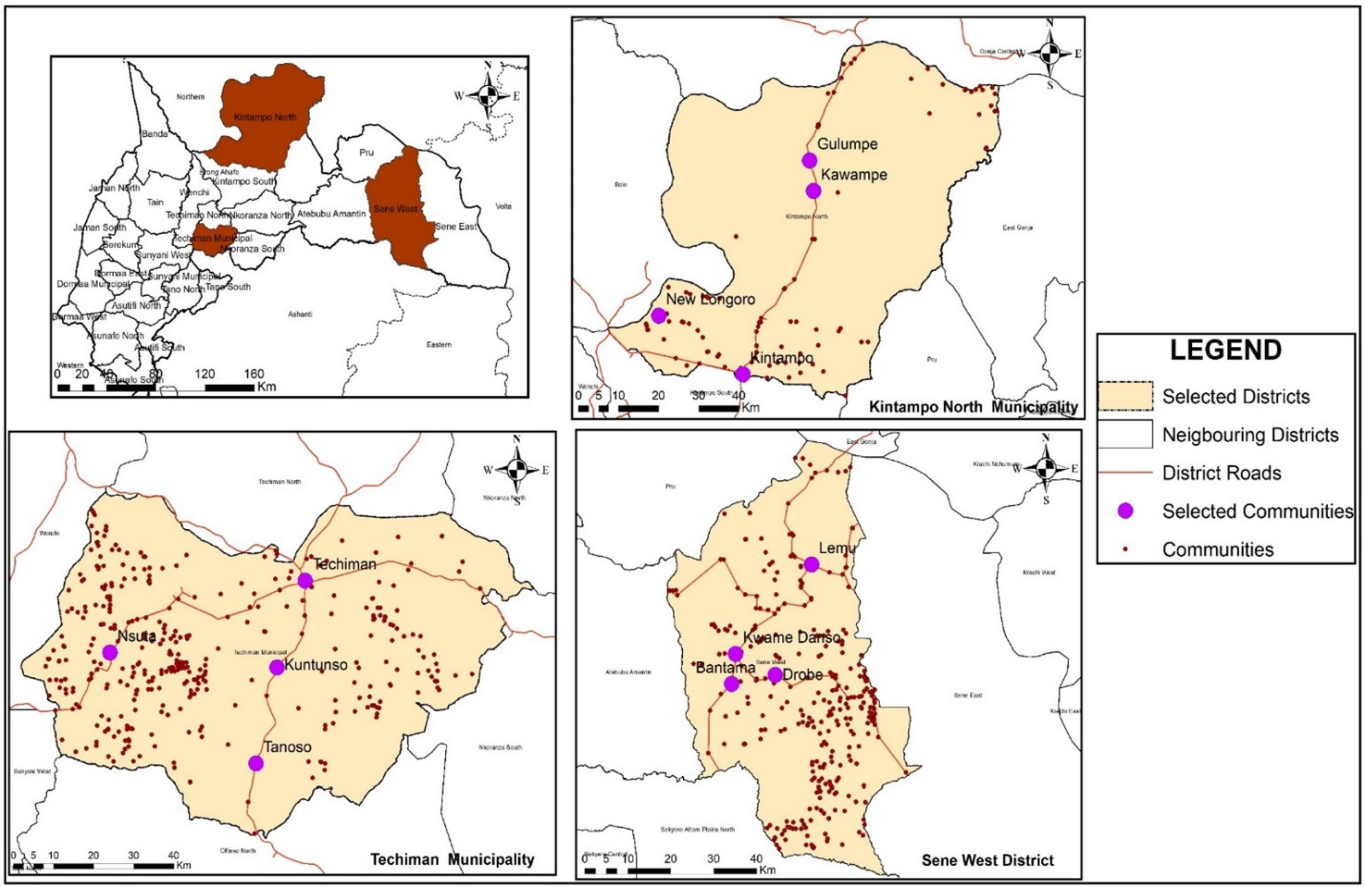
A map of the Brong Ahafo region and selected districts showing communities of data collection.

Purposeful sampling was used to select the three districts (Figure 1). Simple random sampling without replacement (Ahmed, 2009) was used to select three (3) communities in addition to the district capitals from each district. The sampling frame was listed and the manual selection was done for the various districts. The sample size was calculated using Epi Info Software with a confidence level of 95 percent with 80 percent power. Informed consent was obtained from all respondents before the interviews. A total of three to five community key informants were used for the key informant interview (KII). Two separate groups (female and male) of about eight (8) were used for the focus group discussions (FGDs). For individual interviews (II), a total of 684 (684%) respondents were interviewed in the 12 communities.

### 2.3 Key informant interviews (KII) and focused group discussions (FGDs)

For the KII, an average of five people who know the communities and have first‐hand information were used. These were mostly chiefs, community leaders, assemblymen, opinion leaders, and ethnic group leaders. During the FGDs, people who farm, sell, and consume yam were brought together for the discussions. The team worked with the Ministry of Food and Agriculture (MoFA) officers to get the consent of farmers who participated in the study. The participants were allowed to agree or disagree with each other to get an insight into how the group thinks. Facilitation was done with careful wording of the key questions and summarizing to reflect the opinion of the group. Some observations made during the session were noted and included in the report. The discussions included common yams that are grown and the reasons for them, decision‐making in yam production, processing, and marketing, as well as quality characteristics of boiled and pounded yams. Pairwise ranking was used to select quality indicators of boiled and pounded yam for KII, FGDs, and II (RTBFoods, 2018).

### 2.4 Statistical analysis

Data from qualitative interviews were transcribed and coded; themes or categories were developed. SPSS (version 20) was used to analyze quantitative data; frequencies and percentages were calculated on priority quality descriptors of boiled and pounded yam obtained through pairwise ranking. Cramer's *V* (Phi) was used to determine the effect of parameters (sex, age, and ethnic groups) on selected descriptors as well as the differences that exist in the selection of descriptors among respondents. Logistic regression models were then used to describe the data and to explain the relationship between individual descriptors and gender parameters.

### 2.5 Ethical considerations

The key principles in the Humanities and Social Sciences Research Policy of the Kwame Nkrumah University of Science and Technology (HuSSREC, 2018) were employed in this study. The purpose of the research was stated and explained to all who voluntarily participated in this work. Prior to interviews, informed consent was sought from the participants. In the presentation of results, anonymity has been respected.

## 3 RESULTS AND DISCUSSION

### 3.1 Description of communities by key informants

The main occupation of all communities is farming. A few people worked for the government and other nongovernmental agencies, with some involved in artisanal work. Komkombas, Gonja, Mos, Bonos, and Dwans were the dominant ethnic groups, and they coexisted with other minor tribes peacefully. Yam planting and harvesting varied for all the communities. Planting takes a maximum of 5 months, that is, from January to April/May, and also from September to December. Harvesting is, however, dependent on the planting date and varies from July to December. Most (on average, 85 percent) of the people in all of the districts studied were yam producers as well as consumers. In the Techiman East District, while farmers generally produced yams for food, the Bonos produced them for food, while the Northerners, who were known to have larger farms, cultivated them to sell. About 95% of the people in Techiman East farm yams, but not on a large scale (1 acre). *Dioscorea alata* is not normally grown in the Techiman East District because they prefer varieties such as *asobayere* and *dorben* (*D. rotundata*) to *matches* and *akaba* (*D. alata*). In the Kintampo North District, three communities estimated 100 percent for yam cultivation, except New Longoro, with an 80 percent estimate. Larger farms are owned mainly by young men (18–35 years) and children aged 15–16 years (3.2 percent) are said to own yam farms. In Sene West, all (100%) of the farmers were growing yams, with only a few (about 10%) doing mixed cropping for food only. All varieties could be used for *ampesi* or *fufu,* depending on the maturity. The key informants noted that people are buying and consuming more yams than before in their communities. The increase in consumption was linked to the fact that they had a lot of yams. The respondents unanimously admitted, “we grow yams a lot and if sales are bad, we have to eat our yams.” Generally, yams are cultivated, particularly for food and income, in all the districts.

The results from the key informants give a general overview of yam growing and consumption in the communities. It also provides information on preferences for quality descriptors for varieties they grow and consume. According to the key informants, *D. alata* is grown regularly during the growth cycle as observed by Mignouna et al. (2014a, 2014b). The increased consumption is linked to the fact that they produce a lot of yams. From the interactions, the main reasons for yam cultivation are income and household food supply, as reported by Sanginga and Mbabu (2015). The key informants further noted that taste and moldability are the food quality expectations for *ampesi* and *fufu* consumers, respectively. Meanwhile, Aidoo (2009) also found that taste is the single most important factor that determines the variety to be purchased and consumed by urban consumers. Thus the preference for taste, since the growing communities also produce for urban consumers.

Furthermore, knowledge of varieties helps them in their selection, but they added, “the money available will determine the variety to buy.” In Kintampo North and Sene West Districts, people eat *fufu* made from yam only. However, in the Techiman East District, the key informants said people in their communities prefer composite yam and cassava (80:20 percent, respectively) *fufu*. Additionally, they would rather sell cassava, plantain, and cocoyam to purchase good yam varieties like *pona* and *dorben* for *fufu*. This implies yam is a chief crop in this district.

The food quality of yam, especially for *ampesi,* was related to whether it was grown with fertilizer or not. The respondents noted that “yams grown without fertilizer are tastier than those grown with fertilizer.” Similar observations were made by Tiama et al. (2018), who found that tubers untreated with fertilizer had good organoleptic quality, which contrasts with work done by Kouakou et al. (2012), who reported that the organoleptic quality of tubers subjected to mineral inputs was not modified. These varying observations could be attributed to the quantity of inputs added.

### 3.2 Focus group discussions on quality descriptors of yam

**FIGURE 2:**
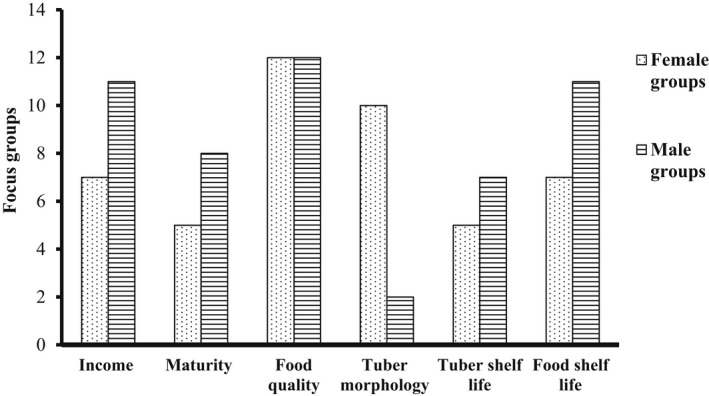
Sex‐based themes from “what a good variety would be like”

Two groups of males and females in each community (12 female and 12 male groups in 3 districts) were separately asked to describe the qualities of generally good yam variety after which they were asked to tell us the most important qualities. From Figure 2, all the groups voted for the food quality more than the other categories mentioned—income, maturity, tuber morphology, tuber, and food shelf life. Males prioritized income‐generating varieties than women. The male groups were concerned more about early maturing and income‐generating varieties while females rated tuber morphological characteristics very high.

The food quality is seen as an important indicator of preference for yams meant for *fufu* and *ampesi*. The perceptions of quality of yam recorded in this study (Figure 2) were similar to those obtained by Mignouna et al. (2014b) which were agronomic, market, and economics. Males prioritized income‐generating varieties than females. This is because yam is considered a man's crop (Mignouna et al., 2014b), and most agricultural activities are carried by them, hence the need for income to take care of their farms and support their families. Generally, varieties prioritized by both males and females are those that have good eating quality, provide income, and have varying maturity periods. The selection of both early and late maturing varieties is based on ensuring food security in their households throughout the year (Fontana et al., 2010).

For both FGDs and individual interviews, yam varieties usually grown or purchased for food include *pona, kununku, afebetua, akaba, matches, labariko, dente, akwa, dorben,* and *asobayere*. For these varieties, numerous reasons were given for their preference. Pairwise ranking was employed to obtain both common and other factors specific to only females or males (Table 1). The preference by males for varieties with good postharvest life aligns with the findings generalized for all yam consumers by Zannou et al. (2015). It also conforms to the global need for improved shelf life of yam tubers (Frossard et al., 2017). Females had varying reasons for their choice of yam variety. Among them is “variety,” which is difficult to become extinct. They explained that “varieties like *akwa* are able to stay on the farmland for a very long time, even after you have stopped planting”. Also, varieties with multiple usages (various yam‐based foods) and those that are easy to cultivate are prioritized by females. This means that any piece of the tuber must be usable as a planting material and all should mature at about the same time regardless of planting time. Females prefer cultivating *D. alata* varieties because they have such characteristics, according to this description (Soibam et al., 2017).

**TABLE 1:** Sex‐based factors affecting the selection of yam variety for cultivation

Female	Male	Factors common to females and males
Good yield	Good postharvest life	Food quality
Easy to cultivate		Marketability
Economical		Maturity
Multiple usage		
Difficult to become extinct		
Accessible planting material		

### 3.3 Household heads and influence on decision making

**FIGURE 3:**
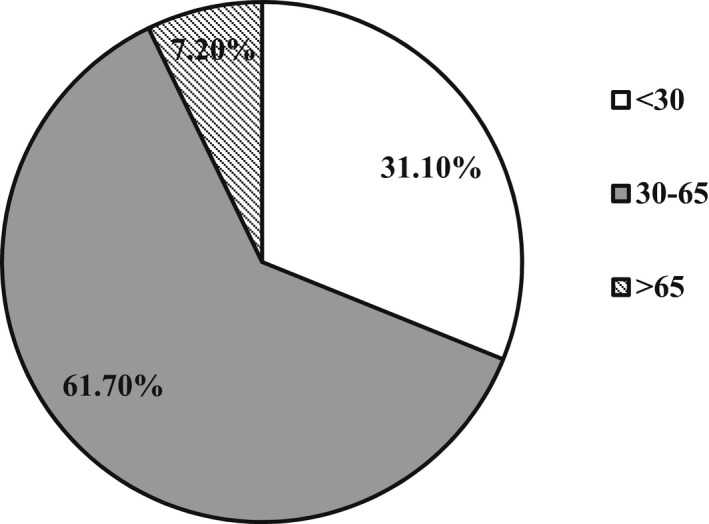
Respondents’ relationship with household heads

**FIGURE 4:**
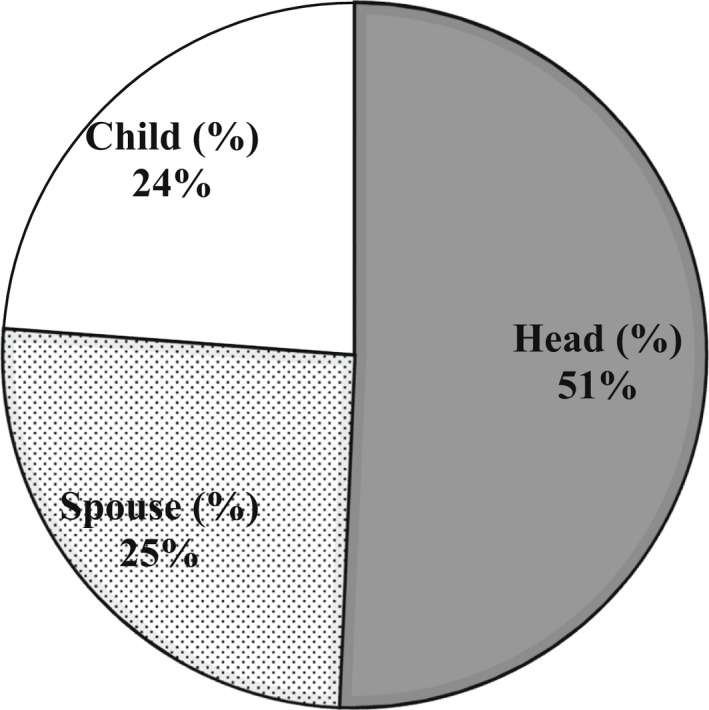
Focused groups’ perception of descriptors of quality of boiled (A) and pounded (B) yam

Majority of individuals interviewed were within the age group 30‐65 years (Figure 3). In Figure 4, the relationships existing between household heads and respondents are shown. Work done by Doss and Morris (2001) shows that male‐ and female‐headed households have different constraints that make them take decisions differently. From the survey, the “head of family” is an individual who is part of the family and gives directives on the use of the family resources. That person, regardless of the amount of time spent with the household throughout the year, still remains the head. The head is not necessarily the husband of the home. It could be the brother or mother of a husband. It could also be the elderly person in the home, whether male or female. The total number of respondents was 195, with 52 divorced or widowed (i.e., 29 percent of the total population). The proportion of females in this category who were household heads, however, was only 2% (Table 1).

Figure 3 shows the relationships existing between household heads and respondents. Work done by Doss and Morris (2001) shows that male‐ and female‐headed households have different constraints that make them take decisions differently. From the survey, the “head of family” is an individual who is part of the family and gives directives on the use of the family resources. That person, regardless of the amount of time spent with the household throughout the year, still remains the head. The head is not necessarily the husband of the home. It could be the brother or mother of a husband. It could also be the elderly person in the home, whether male or female. The total number of respondents was 195, with 52 divorced or widowed (i.e., 29 percent of the total population). The proportion of females in this category who were household heads, however, was only 2% (Table 1).

**FIGURE 5:**
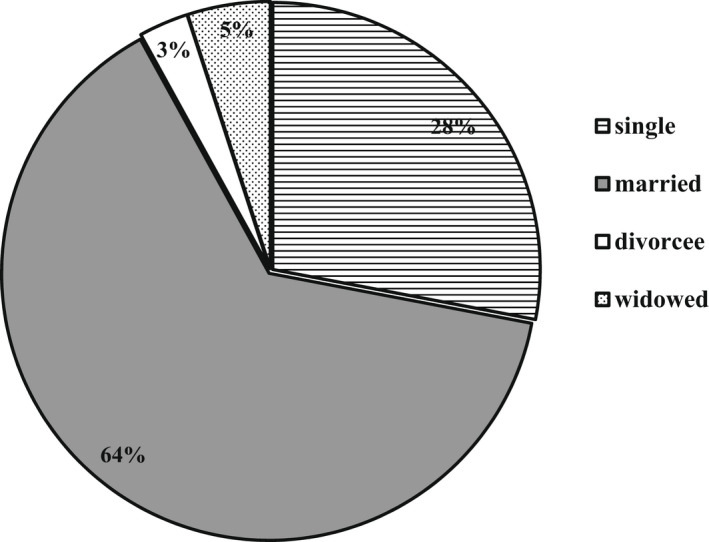
Marital status of individual respondents

From Table 2, the male is generally in charge of planting, marketing, and processing activities. Only 6.6 percent of females are in charge of proceeds, despite the fact that 19 percent are involved in yam planting or own personal farms. From Figure 3, household heads (tradition) play a key role in decision making. Interactions with females who were either divorced and/ widowed (Figure 5) showed that they were not automatically the heads of their homes. These have implications for the choice of yam for processing, which subsequently affects the type of yam‐based food to prepare. Subsequently, the quantity and variety of yams to be processed are mostly determined by the head of the home. The findings show that being single and farming does not make one in charge of marketing and processing activities.

**TABLE 2:** Gender patterns in yam cultivation, marketing, and processing

	Planting (%)	Marketing (%)	Processing (%)	Person in charge of proceeds (%)
Female	130 (19)	84 (12.3)	127 (18.6)	45 (6.6)
Male	253 (37)	249 (36.5)	228 (33.4)	567 (83)
Whole family	114 (17)	130 (19.2)	152 (22.3)	98 (14.4)
N/A	185 (27)	219 (32)	175 (25.7)	28 (4.0)

*Note*: N/A: respondents are not involved in any of the activities mentioned.

### 3.4 Sex‐based FGDs on quality descriptors of raw and processed yam

The respondents believe that the two most important characteristics for yams that have high processing ability are ones that “do not brown” and are “not watery” (Table 3). *D. alata* yam varieties are highly susceptible to browning and have high moisture content. However, they added that few *D. rotundata* varieties also undergo browning. In general, browning is a processing quality that is highly detestable by both female and male groups (14 groups). This was followed closely by the moisture content of the yam (10 groups). Enzymatic browning in yam, caused by the oxidation of phenols by polyphenol oxidases and peroxidases (Graham‐Acquaah et al., 2014), is disliked by farmers, processors, and consumers. The high moisture content is implicative of low dry matter and has a direct effect on its processability and affects the texture of yam‐based foods. However, the moisture decreases with storage (Wireko‐Manu et al., 2013).

**TABLE 3:** Sex‐based preferences of characteristics that show processability

Examples of characteristics of processability	Most important	Female groups	Male groups	Total
Not rotten, smooth skin, does not brown during peeling or after peeling, less watery, no bruises, maturity (sogginess when cut and color), ease of peeling, nice shape, no holes	Not watery Not brown	4 8	6 6	10 14

Figure 4 below (a & b) shows the most important attributes for boiled and pounded yam using pairwise ranking. Only the prioritized attributes are reported for all the FGDs. Generally, of the total scores per attribute, taste is the number one quality attribute for boiled yam (21 groups). In addition to this, males wanted “heavy” and moderately hard yams (9 groups), while females were interested in the aroma (10 groups) of *ampesi*. Although some female groups (4) noted that the whiteness of processed yam was important to them, the color was not as important to most groups (9) as the taste and aroma. For *fufu*, the most important quality parameter for all the FGDs is moldability (17 groups). Smoothness or the absence of lumps (9 groups) and stretchability (9 groups) ranked as second and third priorities, respectively. The keeping quality/shelf life is of more importance to the males (6 groups) than the females (1 group), and the females (7 groups) liked varieties that gave stretchy *fufu*. The males liked “moderately hard” yam varieties. It was explained that “if the yam is sweet and aromatic, its color will not deter people from eating it”.

### 3.5 Participants' characteristics of individuals interviewed

**FIGURE 6:**
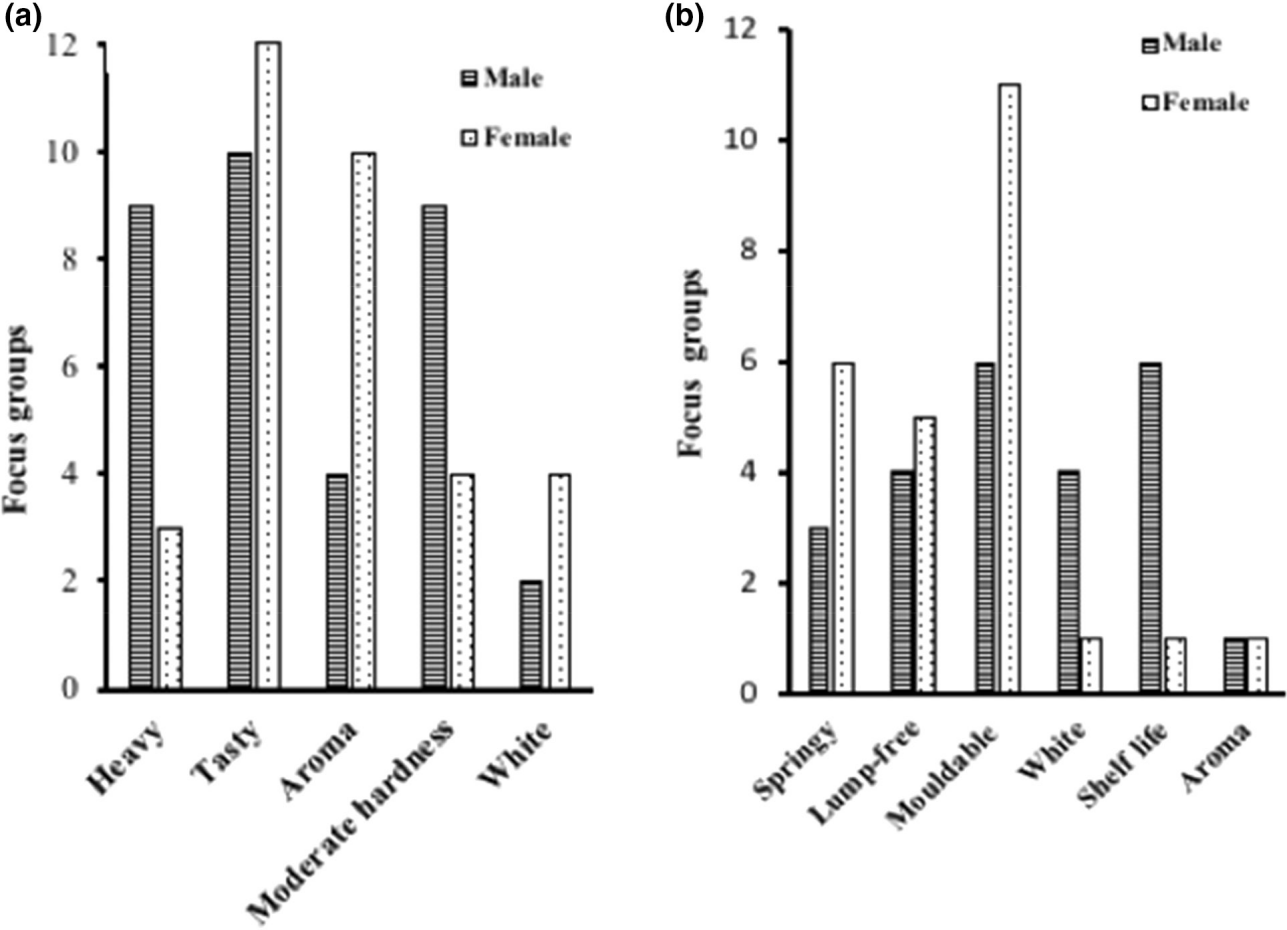
Age groups of individual respondents.

Out of the 684 individuals interviewed, females and males made up 44.6 percent and 55.4 percent of the total. The major ethnic groups identified are grouped in Table 4. The ages and marital status of respondents are presented in Figures 5 and 6. The main religious groups identified are Christianity (67.6 percent), Islam (26.8 percent), and traditionalists (1.3 percent), with 4.3 percent belonging to no religion.

**TABLE 4:** Ethnic groups identified in the individual survey

Major groups	%	Ethnic groups making up major group (%)
Wiase	7.5	
Mos	5.4	
Dwan	19.3	
Akans	32.3	Bono (25), Asante (6.7), Fante (3.8)
Northerner	35.5	Dagbani (2.2) Gonja (18.5), Dagarti (2.5), Sisala (1.8), Kokomba (4.2), Wala (1.0), Bimbila (2.0) Dagomba (3.3)

### 3.6 Preprocessing recognition of yam quality and reasons for selecting yam varieties

Table 5 gives the coded and thematic responses on the characteristics (physicochemical, technological, and postharvest) that help with recognizing quality yam before processing. Participants who stated priorities for the characteristics have been used in the calculations. Overall, the physicochemical characteristics were the most important preselection criteria used by consumers, followed by the technological (knowledge of variety) and postharvest characteristics.

**TABLE 5:** Major themes from characteristics that make a variety suitable food

Examples of codes	Family or theme	Pounded (%)	Boiled (%)
Easy peeling, low water, color of flesh, sliminess, starch content	Physicochemical	211 (42.3)	176 (41.7)
Variety (i.e., knowledge of variety)	Technological	187 (37.50)	148 (35.1)
Uneven, rough, hairy, no holes, skin smoothness, no deformities, size, cracks, color of skin	Postharvest characteristic	101 (20.2)	98 (23.2)
Total		499 (100)	422 (100)

### 3.7 Descriptors of the quality of boiled (*ampesi*) and pounded yam (*fufu*) by individual respondents

Table 6 shows the quality descriptors of boiled and pounded yam from the perspective of the individuals. For boiled yam, a higher percentage of individual respondents (51.5 percent) mentioned sweet/taste as the number one quality index, followed by mealiness (27.8 percent), aroma (12.3 percent), and color (8.5 percent). Moldability is also a key quality descriptor of pounded yam (55.7 percent). Smoothness ranked second (18.7 percent), followed by color (12.3 percent) and stretchability (7.5 percent).

**TABLE 6:** Descriptors of quality of boiled and pounded yam by individual respondents

Quality descriptor	*Ampesi*		*Fufu*	
	Frequency	Percent	Frequency	Percent
Aroma	84	12.3	–	–
Color	58	8.5	–	–
Mealy	190	27.8	–	–
Sweet	352	51.5	–	–
Total	684	100.0	–	–
Color	–	–	84	12.3
Good keeping quality	–	–	21	3.1
Heavy	–	–	19	2.8
Moldable	–	–	381	55.7
Smooth	–	–	128	18.7
Springy	–	–	51	7.5
Total	–	–	684	100.0

Results from individual interviews validated and elaborated the responses from the FGDs, authenticating the findings from the research. Sweet taste, mealiness, and aroma were, respectively, the first, second, and third priorities for quality descriptors of boiled yam. Pounded yam preference was based on moldability, smoothness, color, and stretchability. Moldability is the quality of the yam that allows it to be made into a good shape after pounding without disintegrating over time. Smoothness is the lack of lumpiness in a molded yam sample. The stretchability is a quality parameter that defines the ability of the pounded yam to return to its original state after it has been stretched. Although color is an index of quality, it was not a key descriptor of quality for boiled or pounded yam for the people interviewed. Otegbayo et al. (2010) reported similar findings, with color ranking second in terms of consumer descriptor preference for pounded yams. The keeping quality and shelf life of *fufu*, however, are more important to males than females. Females like varieties that give stretchy *fufu,* whereas males prefer moderately hard yam varieties. They explained that these varieties give a higher yield when pounded (i.e., “expand”) and “stay in the body for long hours”. This could be linked to varieties with low moisture content, high starch, or high dry matter content (Otegbayo et al., 2010). However, their preference for varieties with slow digestion could be linked to yams' having high resistant starches (Moran, 2018).

According to the respondents, “the skin color determines the maturity; very dark brown skin indicates yam is fully matured compared to light brown skin.” Soil color, which also imparts color to the skin, has a significant influence on yam quality. It was also noted that “yams grown in white soils are tastier than those grown in red soils.” Soil color is produced by the minerals present; red soils are known to contain iron and aluminum oxides, while white soils contain silicates and salts high in calcium (Hachem et al., 2020). According to Stuart‐Street et al. (2020), calcium is important for taste development by enhancing the formation of sugars. Furthermore, white soils are normally clayey and have a higher cation exchange capacity, which means they can hold more positively charged mineral ions such as potassium and magnesium, making them more readily available to plants (Hachem et al., 2020). The preceding suggests that the calcium and higher cation exchange capacity of white soils help in the development of taste and improve the mineral content of yams grown in them. However, Otegbayo et al. (2010), in their interaction with focus groups, found that the skin color could not predict the food quality of yam.

### 3.8 Effects of gender parameters on descriptors of the quality of boiled and pounded yams

The effects of gender parameters on quality descriptors were studied using Cramer's *V* (Phi). This value gives the measure of the strength of association between two categorical variables; and varies between 0 and 1 without any negative values (Field, 2013). Cramer's *V* is equal to 0 when there is no relationship; >0.05 weak relationship; >0.10 moderate; >0.15 strong; >0.25 very strong (Akoglu, 2018). Significant differences existed among only the ethnic groups (*p* = .000) for boiled yam (Table 7) with Cramer's *V* (Phi) of 0.132. Phi for sex, age, and occupation (0.053, 0.055 and 0.068, respectively) show very little association. Furthermore, gender parameters, such as occupation and age group segmentation of respondents, had no effect on quality descriptors of pounded yam. However, at *p* < .05, significant differences occurred with ethnic groups (*p* = .00) and sex (*p* = .003). The Cramer's *V* of 0.157 and 0.163 for sex and ethnic group show moderate associations between choices for descriptors.

**TABLE 7:** Descriptors of quality of boiled yam as affected by gender parameters

Parameter/Variable	Aroma (%)	Color (%)	Mealy (%)	Sweet (%)	Total (%)	Chi‐Square			Effect size
						Value	Df	*p*‐Value	Phi/Cramer's *V*
Sex									
Male	49 (12.9)	34 (9.0)	110 (29.0)	186 (49.1)	379 (100)	1.948	3	.583	0.053
Female	35 (11.5)	24 (7.9)	80 (26.2)	166 (54.4)	305 (100)				
Total	84 (12.3)	58	190	352	684				
Age									
<30 years	27 (12.7)	17 (8.0)	60 (28.2)	109 (51.2)	213 (100)	4.081	6	.666	0.055
30–65 years	49 (11.6)	37 (8.8)	122 (28.9)	214 (50.7)	422 (100)				
>65	8 (16.3)	4 (8.2)	8 (16.3)	29 (59.2)	49 (100)				
Total	84 (12.3)	58 (8.5)	190 (27.8)	352 (51.5)	684 (100)				
Ethnic group									
Akan	24 (10.9)	24 (10.9)	63 (28.5)	110 (49.8)	221 (100)	35.90	12	.000	0.132
Dwan	26 (19.7)	5 (3.8)	31 (23.5)	70 (53.0)	132 (100)				
Mo	3 (7.1)	4 (10.8)	5 (13.5)	25 (67.6)	37 (100)				
Northerner	18 (7.4)	18 (7.4)	79 (32.5)	128 (52.7)	243 (100)				
Wiase	13 (25.5)	7 (13.7)	12 (23.5)	19 (37.3)	51 (100)				
Total	84 (12.3)	58 (8.5)	190 (27.8)	352 (51.5)	684 (100)				
Main occupation									
Artisan	10 (11.8)	9 (10.6)	22 (25.9)	44 (51.8)	85 (100)	9.546	9	.388	0.068
Civil service	6 (12.8)	6 (12.8)	10 (21.3)	25 (53.2)	47 (100)				
Farming	63 (13.2)	33 (6.9)	133 (27.8)	249 (52.1)	478 (100)				
Trading	5 (6.8)	10 (13.5)	25 (33.8)	34 (45.9)	74 (100)				
Total	84 (12.3)	58 (8.5)	190 (27.8)	352 (51.5)	684 (100)				

Logistic regressions (appendix 1, 2, 3, 4) for boiled yam showed that the significance observed with the descriptors among the ethnic groups (Table 7) is due to the differences in preference for the descriptor, aroma. For pounded yam, Akans like highly stretchy *fufu more* than the rest of the ethnic groups (Table 8). In terms of keeping quality, preference by the Wiase ethnic group is significantly different (ExpB = 0.00) from the other ethnic groups. Also, the levels of likeness for the descriptor stretchability for Northerners (*p* = .00) and Mos (*p* = .008) are significantly different from the rest of the ethnic groups with high preference. The preference for highly stretchy *fufu* by Akans over the other ethnic groups may be due to the fact that most of them are used to eating *fufu* made from cassava or a combination of cassava and yam, which increases stretchability.

**TABLE 8:** Descriptors of quality of pounded yam as affected by gender parameters

Parameter variable	Quality descriptors of pounded yam						Total	Chi‐Square			Effect size
	Color	Good keeping	Heavy	Moldable	Smooth	Springy		Value	*Df*	*p*‐Value	Phi/Cramer's *V*
Sex											
Male	43 (11.3)	20 (5.3)	11 (2.9)	218 (57.5)	63 (16.6)	24 (6.3)	379 (100)	18.065	5	.003	0.157
Female	41 (13.4)	1 (0.3)	8 (2.6)	163 (53.4)	65 (21.3)	27 (8.9)	305 (100)				
Total	84 (12.3)	21 (3.1)	19 (2.8)	381 (55.7)	128 (18.7)	51 (7.5)	684 (100)				
Age											
<30 years	30 (14.1)	10 (4.7)	6 (2.8)	112 (52.6)	41 (19.2)	14 (6.6)	213 (100)	6.482	10	.773	0.069
30–65 years	51 (12.1)	9 (2.1)	12 (2.8)	239 (56.6)	78 (18.5)	33 (7.8)	422 (100)				
>65 years	3 (6.1)	2 (4.1)	1 (2.0)	30 (61.2)	9 (18.4)	4 (8.2)	49 (100.)				
Total	84 (12.3)	21 (3.1)	19 (2.8)	381 (55.7)	128 (18.7)	51 (7.5)	684 (100)				
Ethnic group											
Akan	22 (10.0)	6 (2.7)	15 (6.8)	131 (59.3)	38 (17.2)	9 (4.1)	221 (100)	72.477	20	.000	0.163
Dwan	9 (6.8)	7 (5.3)	–	86 (65.2)	28 (21.2)	2 (1.5)	132 (100)				
Mo	6 (16.2)	1 (2.7)	–	20 (54.1)	4 (10.8)	6 (16.2)	37 (100)				
Northerner	36 (14.8)	7 (2.9)	4 (1.4)	115 (47.3)	47 (19.3)	34 (14.0)	243 (100)				
Wiase	11 (21.6)	–	–	29 (56.9)	11 (21.6)	–	51 (100)				
Total	84 (12.3)	21(3.1)	19(2.8)	381 (55.7)	128 (18.7)	51 (7.5)	684 (100)				
Main occupation											
Artisan	12 (14.1)	2 (2.4)	1 (1.2)	40 (47.1)	23 (27.1)	7 (8.2)	85 (100)	14.847	15	.462	0.085
Civil service	8 (17.0)	2 (4.3)	2 (4.3)	23 (48.9)	6 (12.8)	6 (12.8)	47 (100)				
Farming	55 (11.5)	14 (2.9)	13 (2.7)	282 (59.0)	81 (16.9)	33 (6.9)	478 (100)				
Trading	9 (12.2)	3 (4.1)	3 (4.1)	36 (48.6)	18 (24.3)	5 (6.8)	74 (100)				
Total	84 (12.3)	21 (3.1)	19 (2.8)	381 (55.7)	128 (17.7)	51 (7.5)	684 (100)				

### 3.9 Differences in descriptor preferences by urban and rural consumers

**FIGURE 7:**
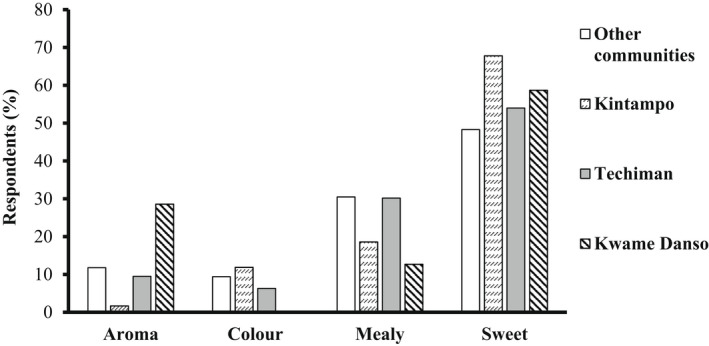
Differences in descriptor preferences among urban and rural consumers for boiled yam.

**FIGURE 8:**
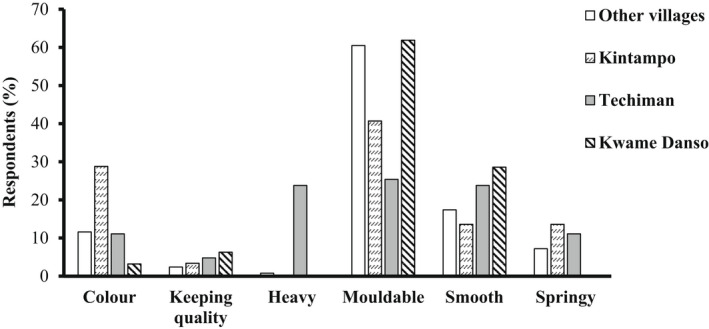
Differences in descriptor preferences among urban and rural consumers for pounded yam.

The figures below (Figures 7 and 8) show the percent contributions of respondents from the rural and urban communities for specific quality attributes of boiled and pounded yam. Significant differences (*p* = .001) are observed in the preferences for descriptors of the quality of boiled yam for 185 respondents from the 3 district capitals and 499 respondents from 9 rural communities (Figure 8). Among the district capitals, there is variability in their liking for the various descriptors (*p* = .00). Similarly, for pounded yam, at *p* = .00, significance is observed among the district capitals and the rural communities in their preferences. Again, the figures depict how highly the sweetness and moldability of boiled and pounded yams are liked by both rural and urban consumers.

Finally, the overall preference for descriptors among rural and urban consumers with *p*‐values (.014 and .004) for boiled and pounded yam, respectively, show significant differences in the liking for the descriptors. The effect size (Cramer's *V*) of 0.125 and 0.238 also depicts strong relationship between the geographical locations and descriptor preferences.

## 4 CONCLUSION

Improving the quality of yam to meet the future needs of consumers requires a systematic approach in which gender disaggregation of information on quality preferences is key. The study has shown that generally, descriptors for good yam variety include income generation, maturity, food quality, tuber morphology, tuber shelf life, and food shelf life. Food quality is important to males and females and is linked to the variety of yam, the type of soil in which it is grown, and whether or not inorganic fertilizers are used. While females prioritized income‐generating varieties over males, males ranked tuber morphological characteristics high. Secondly, the main processing quality traits include low moisture content and color (white). Males described yams that have a longer digestion time as “heavy yams” and preferred such varieties. The shelf stability of *fufu* is an important descriptor that has been generated from this study. Lastly, the study reveals differences in descriptor preferences among various ethnic groups. Preference for the aroma of *ampesi*, as well as the color and stretchability of *fufu*, differed significantly (*p* .05) among the ethnic groups. These identified relationships in descriptor preferences by sex and ethnicity are moderate to strong (Cramer's *V*). The difference in descriptor preference between urban and rural consumers is significant. Hence, the need to consider these gender parameters in future yam breeding programs. Policies must be formulated to ensure that breeding programs welcome research findings on gender and include them in their work. This may improve the adoption rate of new varieties and has the potential to support food security and nutrition.

## CONFLICT OF INTEREST

All authors have no conflict of interest to declare.

## APPENDIX 1

### 1.1 LOGISTIC REGRESSION FOR DEMOGRAPHIC CHARACTERISTICS ON DESCRIPTOR AROMA (BOILED YAM)

**Table:**

	*B*	SE	Wald	*df*	Sig.	Exp(B)	95% C.I. for EXP(B)	
Female	−0.171	0.242	0.499	1	0.480	0.843	0.525	1.354
Age			1.151	2	0.562			
30–65 years	−0.022	0.268	0.007	1	0.934	0.978	0.578	1.655
>65 years	0.431	0.454	0.901	1	0.343	1.539	0.632	3.746
Ethnic group			20.755	4	0.000			
Dwan	0.69	0.31	4.944	1	0.026	1.993	1.085	3.661
Mo	−0.334	0.644	0.269	1	0.604	0.716	0.203	2.53
Northerner	−0.419	0.329	1.625	1	0.202	0.658	0.346	1.252
Wiase	1.146	0.395	8.415	1	0.004	3.147	1.45	6.827
Occupation			2.448	3	0.485			
Civil service	0.236	0.568	0.172	1	0.678	1.266	0.416	3.854
Farming	0.06	0.378	0.025	1	0.874	1.062	0.506	2.229
Trading	−0.671	0.588	1.304	1	0.254	0.511	0.162	1.617
Constant	−2.066	0.42	24.15	1	0.000	0.127		
b. Variable(s) entered on step 1: Sex, Age, ethnic_group, Occupation.								

## APPENDIX 2

### 2.1 LOGISTIC REGRESSION FOR DEMOGRAPHIC CHARACTERISTICS ON DESCRIPTOR COLOR (BOILED YAM)

**Table:**

	*B*	SE	Wald	*df*	Sig.	Exp(B)	95% C.I. for EXP(B)	
Sex(1)	−0.228	0.284	0.646	1	0.421	0.796	0.457	1.388
Age			0.265	2	0.876			
30–65 years	0.159	0.318	0.250	1	0.617	1.172	0.629	2.185
>65 years	0.178	0.592	0.090	1	0.764	1.195	0.375	3.810
Ethnic_group1			7.285	4	0.122			
Dwan	−1.074	0.507	4.484	1	0.034	0.342	0.126	0.923
Mo	0.068	0.579	0.014	1	0.906	1.071	0.344	3.331
Northerner	−0.491	0.330	2.210	1	0.137	0.612	0.320	1.169
Wiase	0.251	0.469	0.288	1	0.592	1.286	0.513	3.222
Occupation			5.234	3	0.155			
Civil service	0.184	0.572	0.104	1	0.747	1.202	0.392	3.691
Farming	−0.528	0.408	1.673	1	0.196	0.590	0.265	1.312
Trading	0.187	0.500	0.139	1	0.709	1.205	0.452	3.212
Constant	−1.808	0.448	16.292	1	0.000	0.164		
Dependent: Color; Variable(s) entered on step 1: Sex, Age, ethnic_group, Occupation.								

## APPENDIX 3

### 3.1 LOGISTIC REGRESSION FOR DEMOGRAPHIC CHARACTERISTICS ON DESCRIPTOR KEEPING QUALITY (POUNDED YAM)

**Table:**

	*B*	SE	Wald	*df*	Sig.	Exp(B)	95% C.I. for EXP(B)	
Female	−2.834	1.031	7.560	1	0.006	0.059	0.008	0.443
Age			2.861	2	0.239			
30–65 years	−0.814	0.489	2.771	1	0.096	0.443	0.170	1.155
>65 years	−0.178	0.815	0.048	1	0.827	0.837	0.169	4.136
Ethnic group			1.824	4	0.768			
Dwan	0.653	0.583	1.253	1	0.263	1.921	0.613	6.027
Mo	0.019	1.127	0.000	1	0.986	1.020	0.112	9.288
Northerner	−0.025	0.575	0.002	1	0.965	0.975	0.316	3.007
Wiase	−17.382	5247.259	0.000	1	0.997	0.000	0.000	
Occupation			1.580	3	0.664			
Civil service	0.870	1.050	0.688	1	0.407	2.388	0.305	18.690
Farming	0.384	0.788	0.237	1	0.626	1.468	0.313	6.879
Trading	1.024	0.960	1.136	1	0.286	2.784	0.424	18.286
Constant	−2.988	0.832	12.910	1	0.000	0.050		

## APPENDIX 4

### 4.1 LOGISTIC REGRESSION FOR DEMOGRAPHIC CHARACTERISTICS ON DESCRIPTOR STRETCHABILITY (POUNDED YAM)

**Table:**

	*B*	SE	Wald	*df*	Sig.	Exp(B)	95% C.I. for EXP(B)	
Female	0.450	0.302	2.226	1	0.136	1.569	0.868	2.834
Age (Ref.: < 30 years)			0.231	2	0.891			
30–65 years	0.162	0.348	0.216	1	0.642	1.175	0.595	2.323
>65 years	0.184	0.611	0.090	1	0.764	1.202	0.363	3.982
ethnic_group (Ref.: Akan)			21.331	4	0.000			
Dwan	−0.989	0.791	1.563	1	0.211	0.372	0.079	1.753
Mo	1.515	0.568	7.118	1	0.008	4.548	1.495	13.840
Northerner	1.360	0.390	12.182	1	0.000	3.897	1.816	8.366
Wiase	−18.026	5603.175	0.000	1	0.997	0.000	0.000	
Occupation Ref.: Artisan)			0.925	3	0.819			
Civil service	0.365	0.609	0.359	1	0.549	1.440	0.436	4.754
Farming	−0.079	0.454	0.030	1	0.862	0.924	0.380	2.249
Trading	−0.177	0.627	0.080	1	0.778	0.838	0.245	2.864
Constant	−3.466	0.585	35.111	1	0.000	0.031		
